# Crop Biotechnology and Product Stewardship

**DOI:** 10.1080/21645698.2020.1822133

**Published:** 2020-10-20

**Authors:** Ruth Mbabazi, Muffy Koch, Karim Maredia, Joseph Guenthner

**Affiliations:** a Professor and Director of International Programs, College of Agriculture and Natural Resources, Michigan State University; b Senior Regulatory Affairs Manager, Simplot Plant Sciences, J. R. Simplot Company, Boise, ID, USA; c Professor Emeritus University of Idaho, Moscow, ID, USA

## Abstract

Agricultural biotechnology is enhancing agricultural productivity, food security, and livelihoods globally. Some developing countries have established functional biosafety regulatory systems and have commercialized genetically modified (GM) crops. Release of GM crops requires enhanced capacity for regulatory compliance and product stewardship to help ensure sustainable use of biotechnology products. We conducted a survey of 66 stakeholders, mostly from Africa and Asia, in two-week international agricultural biotechnology short courses. Respondents showed knowledge of biotechnology benefits and expressed potential barriers to commercialization. They identified 16 crops in the “pipeline for commercialization.” Stakeholders also shared ideas about how to build capacity for product stewardship. Product stewardship is a concept which requires each person in the product life cycle – innovators, scientists, and technology users, to share responsibility. This paper focuses on adoption of product stewardship for post-release management of GM crops which encompasses trait performance, resistance management, integrated pest management (IPM), good agricultural practices, high-quality seeds and planting material, intellectual property management, labeling, identity preservation, consumer acceptance, and effective marketing.

## Introduction

Modern agriculture strongly relies on the application of science and technology. Scientific innovations in the fields of biology, chemistry, and engineering have contributed to the advancement of a highly productive and technologically innovative modern agricultural sector in the western world. Technologies generated using the application of science include modern plant breeding techniques and products and genetically modified (GM) products and crops. These technologies have been globally shared to enhance crop breeding programs. Release of GM crops requires enhanced capacity for regulatory compliance and product stewardship to help ensure sustainable use of biotechnology products.^1^ The high cost of generating these innovations and modern technologies, coupled with the unique characteristics of technology transfer between private and public research institutions, has led to the requirement for responsible use and management of these technologies by the breeders and innovators to ensure not only a return on investment but also product integrity along the product cycle.

Improved crop varieties derived using GM techniques have many advantages. Of interest to farmers are GM crops with enhanced input traits, such as insect resistance, herbicide tolerance, and resistance to environmental stresses, such as drought and nutrient-enhanced crops.^2^ With the development of improved crop varieties and seeds comes standardization and certification to enter the global market.^3^ These requirements necessitate that those involved in the product life cycle share responsibility to ensure sustainable use of the product, hence product stewardship. The Excellence Through Stewardship (ETS) is a global industry-coordinated organization that promotes the adoption of stewardship programs and quality management systems for the full life cycle of biotechnology-derived plant products. The ETS programs promote best practices for developing and using GM crops, defines stewardship as the responsible management of a product.^4^ A broad definition was provided by,^5^who defined product stewardship as “the legal, ethical, and moral obligation to assess products and technologies to ensure that they are safe as well as socially and environmentally responsible.” Both definitions place the responsibility of product stewardship on the developer. Developers, both public and private, implement stewardship programs to ensure compliance with regulations, maintain control and quality of their seeds and planting materials, and ensure that the products sustain their identity and performance, and stay in markets where they are approved.

## Product Stewardship Application for Crop Biotechnology

As technologies advance to the global market, the plant biotechnology industry has continued to improve and support responsible use and management of its products. Industry has set stewardship requirements as mandatory for potential licensees^6–8^ and large private companies through ETS affiliation have streamlined their stewardship best management practices to ensure sustainable use of their technologies. Stewardship requires assessment of the potential impact of a trait, product or technology on human health and the environment. It also includes specific actions to protect the performance of the traits and planting material, to ensure a successful product use, and a return on investment. Stewardship programs can help ensure product quality, provide sustainable access for farmers, enhance consumer confidence, promote adoption, and enhance regulatory compliance for biotechnology crops in developing countries. Components of a good stewardship program for biotechnology crops include identity preservation, trait performance, resistance management, integrated pest management (IPM), good agricultural practices, high-quality seeds and planting material, consumer acceptance, marketing, labeling, and intellectual property management.^1^^,4^

Although stewardship has been used widely in the United States (U.S.) agriculture, the concept is relatively new to developing countries. African Agricultural Technology Foundation (AATF), a member of ETS has successfully demonstrated the benefits of stewardship programs with conventional crops where the quality of planting material and crop performance are monitored and discussed with regulators, researchers, and farmers.^9^ To support the establishment and implementation of stewardship for modern biotechnology products, countries in Africa will require robust regulatory systems to guide access to biotechnology products as well as provide a framework that protects and manages intellectual property. Stewardship begins when a GM crop is conceptualized. ETS guidelines have been developed for the full life cycle of any agricultural plant. The life cycle approach involves all processes from research and discovery; product development; seed or plant production; marketing and distribution; crop production; crop utilization, and product discontinuation. In addition, Africa-specific stewardship materials for biotechnology crops have been developed by Forum for Agricultural Research in Africa.^1,4^

### Stewardship Implementation: Case Studies

#### StarLink Maize

Product stewardship is critical for successful commercialization of biotechnology crops. For example, StarLink maize failed to reach its commercial potential because of inadequate stewardship. StarLink was approved in the U.S. for animal feed^10^ but was awaiting additional allergenicity data before getting food use approval. In the interim, the company grew and distributed StarLink under contract for animal feed but were unable to keep it segregated in storage in the U.S. Inadequate product stewardship allowed some StarLink maize to move into food market channels. In 2000 traces of StarLink DNA were found in taco shells and other maize food products. The company had to withdraw StarLink from all markets at considerable financial and reputational costs.

#### Wheat

Wheat provides another example. A company had conducted research on a herbicide tolerant wheat event but chose not to commercialize this event. Even though the event was not on the market, it was found growing on a farm in Oregon in 2013. The negative reaction on the U.S. wheat export market caused serious economic damage to the wheat industry.

In these examples, technology developers failed to keep control of commercial harvests and regulated planting material, respectively. Failures like these could be caused by human error, inadequate training, unintended consequences of company policy or government regulations, sabotage, animal seed dispersal, or other possible causes.^11,12^

Since the StarLink debacle, some trade associations, including the American Soybean Association, have stepped up to help make closed-loop identity preservation standard to reduce liability^11,13^ says that identity preservation is cumbersome and expensive, but necessary to prevent unwanted mixtures of seed or harvests. According to,^14^ widespread planting of some GM crops, along with market opportunities for non-GM products, “… requires the establishment of identity preservation and segregation systems in which traceability and testing are cornerstones …”

As biotechnology crops become established in developing countries, stewardship has become prominent in regulatory compliance training programs.^15^ The issues of product identity preservation, trait performance, documentation and labeling, and product or trait traceability along the product development cycle have been emphasized by product developers to ensure regulatory compliance and business success.^1^ In Africa, Bt. cotton, Bt. maize, and drought tolerant maize are grown by smallholder farmers, and Bt. cowpea is expected to be commercialized soon. In Asia Bt. maize, Bt. cotton and Bt. brinjal/eggplant have been commercialized, golden rice is pending approval, and late blight potato is in field trials. This paper presents results from a biotechnology stewardship survey of developing country participants at an international biotechnology and biosafety short course.

## Methods

The World Technology Access Program (WorldTAP) in the College of Agriculture and Natural Resources at Michigan State University (MSU) organizes annual two-week international short courses in Agricultural Biotechnology and Biosafety at the MSU Campus with visits to other locations in the U.S. Each year around 20–25 international participants from all over the world attend these courses.

Participants of the MSU training courses represent a diverse group of stakeholders including biotechnology regulators, policymakers, scientists, academic specialists, journalists, lawyers, as well as representatives of media, non-government organizations, and private sector. The information on product stewardship included in this paper was provided as part of a need’s assessment survey completed by 66 participants from the training courses offered in 2016 and 2017. The survey was conducted using a survey instrument/questionnaire.

The objectives for the stewardship part of the survey were to:

1. Identify and rank barriers to moving biotechnology crops beyond confined field trials (CFT)
2. Identify biotechnology crops “in the pipeline for general release/commercialization”
3. Seek participants ideas about stewardship

## Results

Sixty-six short course participants from 25 countries completed the survey. Indonesia was the country with the largest number of participants at 9. Next was Ethiopia with 7, followed by China with 6. There were 32 participants from 12 countries in Africa. Asia was represented by 20 participants from 5 countries. The remaining 14 participants came from Europe and the Americas.

More than half (42) of the participants identified themselves as scientists. The next most frequently listed profession was academia with 19, followed by regulators at 15. One of the two participants from private industry was the CEO of a cotton company. The participants represented public, private, and non-government organizations responsible for different elements of product stewardship from product development perspectives.

### Opinions about Commercialization

The survey asked participants to rank eight possible socio-economic benefits of biotechnology crops in their own country. The answers were recorded on a 5-point Likert scale^16^ ranging from a value of 1 for “definitely not beneficial” to 5 for “definitely beneficial.” All eight aspects of socio-economic benefits of biotechnology crops were ranked on the beneficial side of a neutral 3 (Table 1).

**Table 1.** Ranking of biotechnology crop benefits

Rank	Benefit	Average
1	Higher farm revenue	4.4
2	Lower farm production costs	4.3
3	Reduced risk in farming	4.2
4	Improved food quality	4.0
5	Lower food price	3.9
6	Higher standards of living in rural areas	3.8
7	More interest in farming careers	3.4
8	More opportunities for women in agriculture	3.3

Likert scale: 1=definitely not beneficial, 2=not beneficial, 3=neutral, 4 beneficial, 5=definitely beneficial.

Ranked first was “higher farm revenue.” The top three benefits were all farm related. The next two – food quality and price – were about consumer benefits. The combination of benefits to producers, consumers, and the community suggests that participants in general had a positive opinion of agricultural biotechnology benefits. These benefits can be enhanced through maintaining control and quality of seeds and planting materials, product integrity, and keeping products in markets where they are approved.^1,4,6–8^

Another survey question was about the status of GM crop development in the respondent’s country. Twenty-five percent of the respondents said that their country was in the confined field trials (CFT) stage of biotechnology development. Other stages were laboratory research 19%, multi-location field trials (MLT) 18%, greenhouse trials 15%, imports 14%, and commercial production 9%.

We probed for more information about biotech crop development with this question: *“Rank on a scale of 1 to 5 (1 being least important to 5 being most important), the significance of the following constraints/barriers to moving biotech crops beyond the confined field trials to product commercialization and/or general release.”*

We obtained a ranking of eight barriers (Table 2). The top-ranked barrier was regulatory hurdles, followed by public acceptance. Socio-economic constraints ranked third, followed by cost of commercialization, which suggests that economic barriers slow or shut down the pipeline in some countries. Effectiveness of the trait was the lowest ranked barrier.

**Table 2.** Ranking of barriers to moving beyond confined field trials

Rank	Benefit	Average
1	Regulatory hurdles	4.0
2	Limited public acceptance	3.8
3	Socio-economic constraints	3.5
4	Cost of commercialization	3.5
5	Fear of corporate monopolies	3.3
6	Limited capacity for regulatory decision making	3.2
7	Poor access to GM technology	2.8
8	Effectiveness of trait	2.7

Likert scale: 1=least important, 2=less important, 3=neutral, 4=more important, 5=most important.

We asked participants if there were crops in the pipeline for commercialization in their countries. They identified 16 crops at the time of the surveys in 2016 and 2017. We used a database from the International Service for the Acquisition of Agri-biotech Applications^17^ to observe how those 16 crops were moving through the pipeline.

We found that seven of the 16 crops gained GM event approval in at least one country during the 2016–2020 period (Table 3). Cowpea had an event approved for the first time, making it through the regulatory process in Nigeria. Four of the survey participants came from Nigeria.

**Table 3.** Crops identified by the participants as “in pipeline for commercialization.”

		Events approved^b^		
#	Crop^a^	pre-2016	2016–20	Countries^1^ approving events in 2016–2020^c^
1	Banana	0	0	
2	Cassava	0	0	
3	Corn/maize	116	80	ARG, AUS, *BRA*, CAN, *CHN*, COL, *ETH, EU, IDN*, IRN, JPN, KOR, MEX, MYS, *NIG*, NZL, PAK, *PHL*, PRY, SGP, *SWZ, TUR*, TWN, USA, VNM, *ZAF*
4	Cotton	64	26	AUS, *BRA*, CAN, *CHN*, COL, CRI, *ETH, EU*, JPN, KOR, MEX, MYS, *NIG*, NZL, *PHL*, PRY, *SWZ*, TWN, USA
5	Cowpea	0	1	*NIG*
6	Eggplant	1	0	*BGD*
7	Eucalyptus	1	0	*BRA*
8	Mustard	0	0	
9	Papaya	6	0	USA, *CHN*
10	Potato	43	11	ARG, AUS, CAN, JPN, MYS, MEX, NZL, USA
11	Rice	5	2	AUS, CAN, *IDN*, NZL, *PHI*, USA
12	Shallot	0	0	
13	Sorghum	0	0	
14	Soybean	36	27	ARG, AUS, *BRA*, CAN, *CHN*, COL, *EU, IDN*, IRN, JPN, KOR, MEX, MYS, *NIG*, NZL, *PHL*, TWN, SGP, USA, *ZAF*
15	Sugarcane	3	3	*BRA, IND*, CAN, USA
16	Wheat	0	0	
	Totals	275	150	

^a^Crops identified by survey participants.

^b^Source: ISAAA database May 2020; approved for food, feed or cultivation.

^c^Participants’ countries are in italics format.

^1^ ARG – Argentina, AUS – Australia, BGD – Bangladesh, BRA – Brazil, CAN – Canada, CHN – China, COL – Colombia, CRI – Costa Rica, ETH – Ethiopia, EU – European Union, IDN – Indonesia, IRN – Iran (Islamic Republic of), JPN – Japan, KOR – Korea (the Republic of), MEX – Mexico, MYS – Malaysia, NIG – Nigeria, NZL- New Zealand, PAK – Pakistan, PHL – Philippines (the), PRY – Paraguay, SGP – Singapore, SWZ – Eswatini, TUR – Turkey, TWN – Taiwan (Province of China), USA – United States of America (the), VNM – Viet Nam, ZAF – South Africa.

A GM Sugarcane event was approved in Indonesia after the survey. Nine of the survey participants came from that country. The Philippines is another country with survey participants (2) where approval is pending for rice and eggplant. One participant came from Brazil, where a GM sugarcane event was approved after the survey. These results indicate that country progress in biotechnology R&D is a good precursor to the level of understanding of key elements required in the adoption and use of the technology. In this case, participants from countries such as Brazil, Indonesia, and the Philippines were familiar with the concept of product stewardship when compared to participants from countries who did not have advanced R&D in crop biotechnology.

Five of the crops – banana, cassava, mustard, shallot, and sorghum – have not had events approved in any country since the time of the survey.

Overall, there were 426 events approved for the 16 crops to-date. Thirty-five percent (150 events) were approved in the 4–1/2 years from January 2016 to May 2020, suggesting an approval acceleration in recent years. The approval rate is above the average rate of the 16-crops for maize (41%), cowpea (100%), soybean (43%), and sugarcane (50%) were noted to be greater. Table 3 data indicates that some developing countries are contributing to the acceleration.

### Stewardship

**Figure 1.**
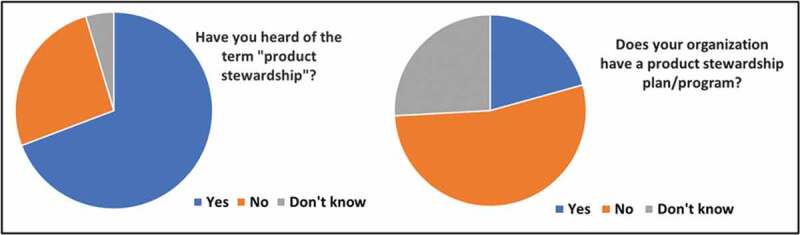
Participants knowledge of stewardship

We asked survey participants several questions about product stewardship. Sixty-nine percent of them answered that they had heard of the term (Figure 1). Twenty-one percent said that their organization has a product stewardship program, but 15% did not know.

The survey asked: what are the key components of a product stewardship program? Participants ranked product safety and product quality as the top two (Table 4). The participants who responded to the survey were mainly from the public sector. Although the participants ranked product safety and product quality as the key areas for product stewardship programs, personal communication with private industry highlighted preventing trade disruptions as one of the key benefits of product stewardship programs which could be derived from several product stewardship components identified by the survey participants, such as product safety, product quality, product marketing and branding, product distribution, product labeling, intellectual property management and licensing, and product identity preservation. Identity preservation and unapproved uses/liability were ranked at the bottom, but they were checked by at least 47 of the 66 participants.

**Table 4.** Key components of product stewardship identified by participants

Rank	Component	Participants checked
1	Product safety	63
2	Product quality	61
3	Monitoring and compliance	59
4	Marketing, branding, sales and distribution	57
5	Product development and testing	56
6	Packaging, transport, storage and disposal	55
7	Product labeling	55
8	Intellectual property management and licensing	54
9	Training	54
10	Identity preservation	53
11	Unapproved uses and liability	47
	I don’t know	6

We asked: what do you think should be the key components of an “identity preservation” program for a GM crop? Worker training about how to prevent mixing of GM with non-GM product was ranked at the top. Second was to require growers and handlers to sign stewardship contracts and to participate in training.

Training was also the top ranked answer to the question: what can be done to build capacity in designing and/or enhancing institutional product stewardship programs? (Table 5). The question was open-ended, so participants answered it in their own words. We sorted those answers into the categories in Table 5.

**Table 5.** Capacity building programs to enhance institutional product stewardship programs

Rank	Category	Number of answers
1	Training	12
2	Education	10
3	Regulations	9
4	Build capacity	5
5	Communications	5
6	Technology	2
7	Identify champions	1
8	Investment	1
9	Labeling	1
10	Network	1
11	Safety	1
12	Other	1

Education was ranked second behind training. Although training and education are both about learning, there are differences. Training is usually undertaken to teach and learn certain skills. Those skills could be the farming practices that minimize the risk of the loss of identity preservation.

Education is generally viewed as developing knowledge and intellect. In the case of product stewardship, education, training, and capacity building could be to develop an understanding and create awareness of why certain farming practices are important to ensure proper use and sustainability of a GM crop.

## Discussion

One of the reasons that public sector biotechnology crops are not successfully commercialized is the gap between developing a new crop and getting planting material into the hands of farmers.^18^ The mandate for commercializing public sector crops is not always clear and the skills and infrastructure needed to implement sustainable seed or vegetative planting material delivery systems are often lacking.^19–21^

Added to this is the stewardship responsibility for maintaining the quality of planting material and managing that biotechnology crops are directed only to the countries where they have been approved. The demand for GM crops in developing countries is expanding. According to,^22^ “Rapid expansion of transgenic crops over the past two decades in the developing world was a result of an intense desire by farmers to adopt these crops irrespective of regulatory roadblocks.”

### Survey Implications

We conducted the survey at the beginning of the short courses. Participants’ in-coming knowledge about stewardship was enhanced during the educational program. We could have conducted the survey at the end of the course, and some answers to questions might have been different. We wanted to get answers from typical participants who are knowledgeable but who probably had not attended a similar two-week short course.

Respondents understood the socio-economic value of biotechnology and ranked regulatory hurdles as the top barrier to moving beyond confined field trials. They also identified 16 crops as “in the pipeline for commercialization.” Seven of those crops have had approved events for food, feed, or cultivation. This indicates that for 7 of the 16 crops, despite multiple barriers, there had indeed been movement beyond CFT since the first short course.

Longer-term outlook is for many more crops to enter the pipeline. The Indian Ministry of Environment, Forests and Climate Change conducted a study in 2016 to learn about GM crops that may enter the pipeline to commercialization in the next 10 y. A survey of public and private experts in India identified 92 plants in the Research & Development pipeline.^23^

Proper stewardship can help move biotechnology crops through the pipeline to successful commercialization. Approval of a crop in a developing country is merely a promise unless it is accompanied by a system that can put biotech seed in the hands of farmers. If high farm revenue, low production costs, and reduced risk are to be achieved, farmers need high-quality seeds and planting materials.

### Access to Quality Seeds and Planting Materials

A primary goal of stewardship is to ensure that new crop planting material is not only high quality but also maintains all the traits promised by the developer. For drought-tolerant maize and insect-resistant brinjal/eggplant, cotton, and cowpea developing countries need reliable seed systems. For late blight potatoes, the planting material is sprouting tubers that should be produced and stored in a way that ensures successful production.

Obtaining regulatory approval is just one step in the stewardship process. While the regulatory review is in process, the applicant can implement the stewardship needed to ensure that the approved planting material can become available for farmers.

Commercial Bt cotton seed in Africa has been provided by private sector partnerships with the cotton milling companies. Drought tolerant maize seeds are provided by public–private partnerships in Africa. It is not yet clear how late blight potato tubers will be marketed in Asia, but it is likely that this planting material will be distributed along the same lines as public sector conventional planting material.

A primary component of stewardship is the provision of insect resistance management that will reduce the likelihood of the target pests developing resistance to the Bt proteins. In the same way, integrated pest management will be used to sustain the late blight protection trait in the potatoes developed for smallholder farmers in Asia.^24^ These resistant management systems^19,25,26^ are sometimes mandated by regulators, but the components are developed and implemented by developers. The resistance management components are specific for different growing environments and need to be incorporated into an overall integrated pest management (IPM) system for each crop.^27^

### Product Stewardship in the Context of African Regulatory Systems

Cotton and maize crops commercialized in Africa were approved in other countries before seeking approval in developing countries. Even with environmental and food safety approvals already obtained, the regulatory burden for subsequent approvals in Africa and Asia are time consuming and expensive. Increased efforts are needed to apply approvals in one country to decisions in neighboring countries with similar environments and socio-economic constraints. The presence of reliable product stewardship enables regulators to address potential socio-economic risks that can be managed by the applicant with appropriate stewardship measures.

As biotechnology science and regulation evolve, enhanced deployment of biotechnology crops becomes essential. A strong product stewardship strategy is important not only for trait preservation but also for effective use and management of biotechnology products.^28^

To design a good stewardship program, it is important to understand each country’s agricultural production and regulatory systems. In most African countries, deployment of public sector crop varieties happens through government supported agricultural extension systems with weak links between research, extension, and farmers. Responsible and effective use of crop protection products is promoted through appropriate advisory services and all stakeholders require training and support.

Most public-private biotechnology crop developers in Africa have established product stewardship programs. Public–private partnerships to commercialize biotechnology crops are setting standards for these programs. However, public sector developers may struggle to set up stewardship because inadequate infrastructure for storage, transport, and distribution, in many countries, may compromise the business plan and product integrity.

A stewardship capacity building program for Africa, Strengthening Capacity for Safe Biotechnology Management in Sub-Saharan Africa (SABIMA), developed stewardship guidelines and training programs appropriate for smallholder and commercial farming on the continent.^29^ The SABIMA case studies for key African crops are used as training materials and guidelines for public sector and private developers of biotechnology crops.^1^

## Conclusion and Way Forward

Sustainable deployment of biotechnology products in Africa requires practical stewardship programs and strong linkages among agricultural research, extension, and farmers. Awareness, education, training, and capacity building for all key stakeholders are critical for effective implementation of product stewardship.

Public–private partnerships could facilitate transfer of stewardship expertise and experiences to public sector projects. Countries in Africa have access to SABIMA training materials and guidelines and can learn from experiences and resources from other countries that have successfully deployed biotechnology crops. Michigan State University’s World Technology Access Program (WorldTAP) provides opportunities for training and capacity enhancement for technology commercialization and product stewardship in developing countries.^30^
